# Robotic omentopexy following sleeve gastrectomy: technique and early outcomes in 65 consecutive patients

**DOI:** 10.1093/jscr/rjag146

**Published:** 2026-03-12

**Authors:** Miljana Vladimirov, Firas Makdesi, Panagiotis Lainas, Carolina Baz, Alejandro Gandsas

**Affiliations:** Department of Surgery, University of OWL-Campus Lippe, Detmold, Germany; Department of Surgery, University of OWL-Campus Lippe, Detmold, Germany; Department of Metabolic & Bariatric Surgery, Metropolitan Hospital, Athens, Greece; Division of Surgery, School of Medicine, European University of Cyprus, Nicosia, Cyprus; Department of Surgery, Luminis Health, Annapolis, MD, United States; Department of Metabolic & Bariatric Surgery, Metropolitan Hospital, Athens, Greece

**Keywords:** sleeve gastrectomy, omentopexy, robotic, bleeding

## Abstract

Staple-line complications after sleeve gastrectomy remain a technical concern. Omentopexy offers an extraluminal support of the gastric sleeve without direct staple-line manipulation. This report shares our experience in using a robotic platform to facilitate and standardized the technique. A retrospective analysis was conducted of patients undergoing robotic sleeve gastrectomy with concomitant omentopexy. Sixty-five patients (64.6% female); median age 47 years, with mean body mass index of 44.7 ± 4.4 kg/m^2^, underwent robotic sleeve gastrectomy with omentopexy. The omentopexy added a mean of 15.5 min to operative time. Median length of stay was 1.3 days, reflecting institutional policy. There were no intraoperative, perioperative, or 30-day complications, readmissions, or reoperations. Robotic omentopexy is a safe, reproducible adjunct to sleeve gastrectomy that adds minimal operative time and provides physiologic extraluminal support. Further studies are needed to assess long-term outcomes.

## Introduction

Staple-line complications following sleeve gastrectomy, particularly bleeding, gastric torsion, and functional narrowing, remain an area of ongoing technical refinement. A variety of reinforcement strategies have been described, including staple-line oversewing, buttressing materials, sealants, and omentopexy. Omentopexy has emerged as a physiologic, extraluminal reinforcement technique, providing external support to the gastric sleeve without direct manipulation of the staple line. Multiple studies have demonstrated that omentopexy may reduce staple-line bleeding, gastric twisting, and postoperative gastrointestinal symptoms compared with non-reinforced sleeves, with complication rates comparable to conventional reinforcement methods and minimal added cost [1–5].

The robotic platform offers specific technical advantages for this maneuver, including enhanced visualization, wristed instrumentation, and precise intracorporeal suturing. These features facilitate controlled and reproducible omentopexy, particularly along the proximal sleeve where exposure and ergonomics can be challenging laparoscopically [6]. The purpose of this report is to describe our experience using a robotic platform to facilitate and standardize the omentopexy technique.

## Series report

The patient is positioned supine, and pneumoperitoneum is established using a Veress needle (Ethicon, Cincinnati, OH, USA) at the left costal margin along the midclavicular line (Palmer’s point). Under direct visualization, three 8-mm robotic trocars and one 12-mm trocar are placed, and the robotic platform (da Vinci Xi Surgical System, Intuitive Surgical, Sunnyvale, CA, USA) is docked. This port configuration provides optimal access to the upper abdomen and facilitates precise intracorporeal suturing, particularly during the omentopexy phase of the operation.

Sleeve gastrectomy is then performed in a standardized manner. Dissection along the greater curvature begins ~4–6 cm proximal to the pylorus and proceeds cephalad toward the left crus. The greater omentum is carefully divided, with complete mobilization of the gastric fundus and systematic division of the short gastric vessels while preserving splenic integrity. Particular attention is paid to releasing posterior gastric attachments to eliminate residual fundal tension, a factor that has been implicated in postoperative sleeve torsion and functional obstruction. Adequate exposure of the angle of His and left crus is ensured to prevent retained fundus and to optimize sleeve geometry.

A 36-French bougie is introduced along the lesser curvature to calibrate the sleeve and ensure uniform gastric diameter. Gastric transection begins ~5 cm from the pylorus and advances proximally toward the fundus using sequential, slightly overlapping staple loads selected according to gastric wall thickness. Throughout stapling, the stapler is maintained parallel to the bougie to avoid sleeve spiraling or narrowing, particularly at the incisura angularis. Prior to each firing, the stapler is gently compressed for several seconds to optimize staple formation and minimize bleeding. The final firing near the angle of His is performed with particular care to ensure complete fundal resection, as residual fundus may adversely affect weight loss outcomes or contribute to postoperative gastroesophageal reflux.

**Figure 1:**
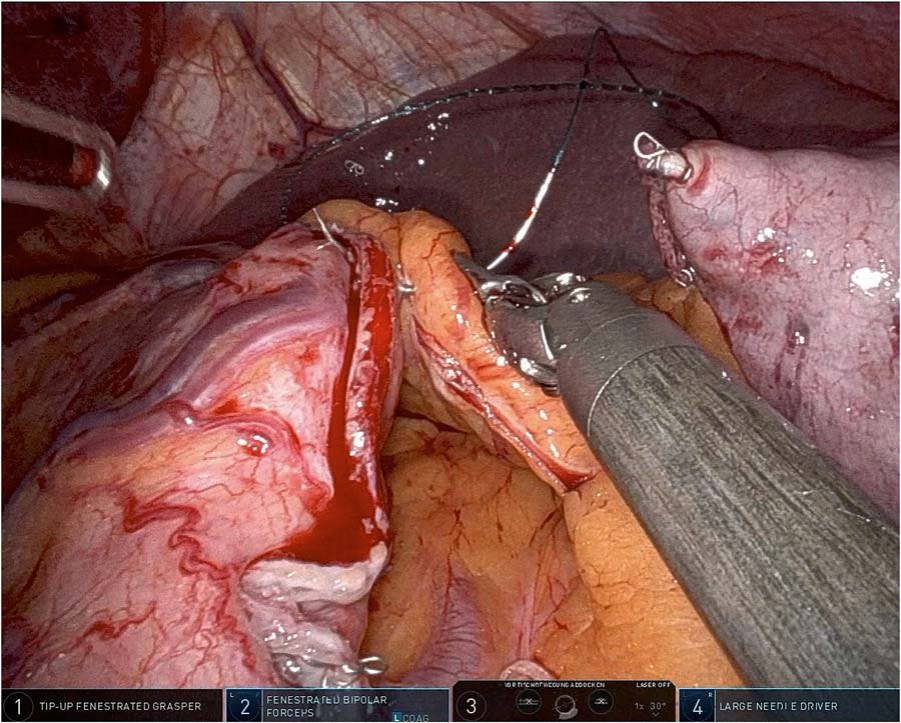
Robotic omentopexy during sleeve gastrectomy.

Following completion of the sleeve, meticulous hemostasis is confirmed, and the integrity of the staple line is assessed using methylene blue instillation. In the absence of leakage, omentopexy is then performed. The greater omentum is sutured to the seromuscular layer adjacent to the staple line using a continuous absorbable barbed suture (3-0 V-Loc™, Medtronic, Minneapolis, MN, USA), beginning proximally at the angle of His and extending distally along the length of the sleeve (Fig. 1).

Using robotic needle drivers, the free edge of the greater omentum is aligned along the length of the staple line without tension. Beginning proximally near the angle of His, the omentum is secured to the seromuscular layer adjacent to the staple line using interrupted or short running absorbable sutures. Sutures are spaced ~1 cm apart and extended distally toward the antrum, typically stopping short of the pylorus. If sutures are spaced ˃1 cm apart, tightening them may increase the risk of sleeve wrinkling and functional obstruction.

This technique mirrors previously described laparoscopic omentopexy approaches while leveraging robotic articulation for improved needle control and tissue handling [1, 2, 4]. Importantly, no full-thickness gastric bites are taken, and the staple line itself is not penetrated.

**Figure 2:**
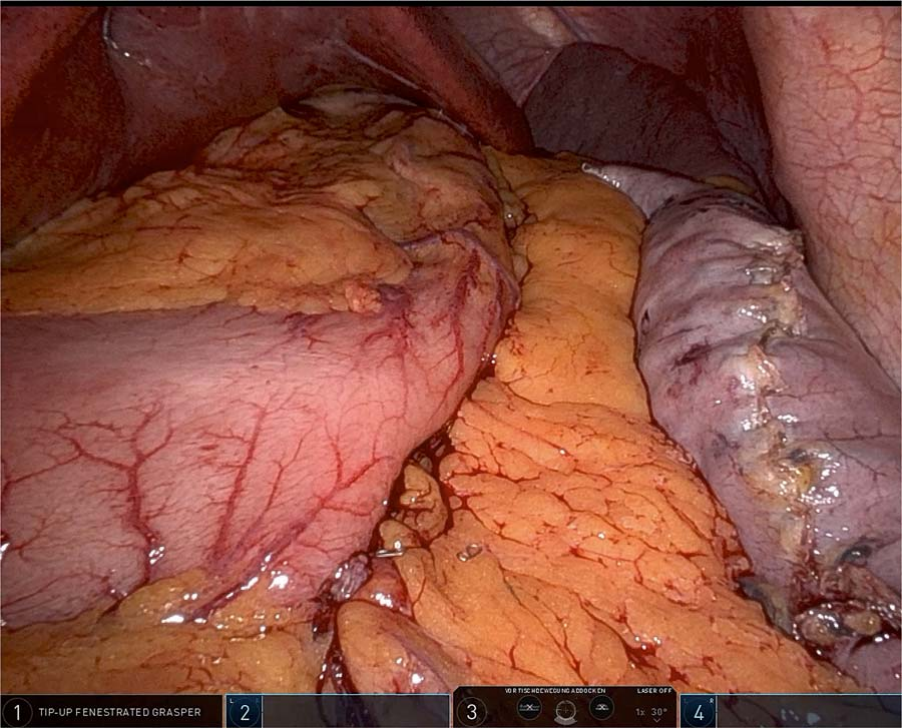
Robotic omentopexy after completion of sleeve gastrectomy.

The omentum is positioned evenly without excessive tension, providing external reinforcement and hemostatic support while preserving sleeve geometry. This maneuver also serves to stabilize the gastric tube and may reduce the risk of postoperative torsion or migration (Fig. 2).

The resected gastric specimen is extracted through the 12-mm trocar site. Fascial closure is performed as indicated, and skin incisions are closed using absorbable sutures.

This technique was performed in 65 patients (64.6% female) with a median age of 41 years and mean body mass index (BMI) of 44.7 ± 4.4 kg/m^2^, who underwent omentopexy at the conclusion of robotic sleeve gastrectomy. The median operative time was 130 min (120–150). The omentopexy component added a mean of 15.5 min. There were no conversions to open surgery and no intraoperative complications (Table 1).

**Table 1:** 

Variable	Value
Age, years (mean ± SD)	47.2 ± 12.1
Female sex, n (%)	42 (64.6%)
BMI, kg/m^2^ (mean ± SD)	44.7 ± 4.4
Operative time, min (median, IQR)	130 (120–150)
Additional omentopexy time, min (mean)	15.5
Length of stay, days (mean ± SD)	1.3 ± 0.6
30-day bleeding, n (%)	0 (0%)
30-day leak, n (%)	0 (0%)
30-day stenosis, n (%)	0 (0%)
30-day readmission, n (%)	0 (0%)
30-day reoperation, n (%)	0 (0%)

Abbreviations: BMI = body mass index; IQR = interquartile range; SD = standard deviation.

The median length of hospital stay was 1.3 ± 0.6 days. This duration reflects institutional and administrative policy within the German healthcare system, where routine postoperative inpatient observation following bariatric surgery is standard practice, rather than being driven by postoperative recovery, complication burden, or need for prolonged clinical monitoring.

There were no perioperative or 30-day postoperative complications, including staple-line leak, postoperative bleeding, gastroesophageal reflux disease, or need for reintervention. No 30-day readmissions, reoperations, or postoperative imaging or endoscopic evaluations were required. This analysis was designed to assess the feasibility and short-term safety of robotic omentopexy rather than long-term clinical or weight-loss outcomes.

## Discussion

Omentopexy functions as a form of external staple-line reinforcement, offering gentle compression and mechanical support that may contribute to improved hemostasis and sleeve stabilization. Randomized and comparative studies looking only at laparoscopic sleeve gastrectomy, have demonstrated lower rates of staple-line bleeding and reduced gastric torsion in sleeves reinforced with omentopexy compared with non-reinforced sleeves [1, 2]. Large cohort studies have further suggested that omentopexy may decrease functional complications such as twisting and postoperative gastrointestinal symptoms without increasing leak rates [3–6].

Unlike traditional staple-line reinforcement techniques, such as oversewing or buttressing materials applied directly to the staple line, omentopexy avoids intraluminal narrowing and preserves sleeve geometry [2, 4]. While recent data suggest that omentopexy may not provide additional benefit when combined with staple-line imbrication, its role as a standalone or alternative reinforcement strategy remains well supported in non-imbricated sleeves [7].

Robotic omentopexy performed at the conclusion of robotic sleeve gastrectomy is a safe and reproducible adjunct that leverages the advantages of the robotic platform to facilitate precise intracorporeal suturing and controlled tissue handling. In this series, the technique was associated with minimal additional operative time and demonstrated excellent short-term safety, with no perioperative or 30-day complications observed. By providing extraluminal support to the gastric sleeve without direct manipulation of the staple line, omentopexy represents a physiologic alternative to traditional reinforcement strategies and may contribute to improved hemostasis and sleeve stabilization. While long-term outcomes remain to be defined, this technique offers a standardized and easily adoptable approach for surgeons performing robotic sleeve gastrectomy.

Omentopexy following robotic sleeve gastrectomy is a feasible adjunct with minimal added operative time and favorable early postoperative outcomes in this consecutive series. Further comparative research is warranted to clarify its impact on long-term clinical outcomes.
